# Exploring the ethical and practical challenges of conducting clinical trials in care home settings

**DOI:** 10.1186/1745-6215-12-S1-A38

**Published:** 2011-12-13

**Authors:** Fiona Wood, Hayley Prout, Arun Acharjya, Jacqueline Nuttall, Kerry Hood, Christopher Butler

**Affiliations:** 1 Department of Primary Care and Public Health, School of Medicine, Cardiff University. Heath Park, Cardiff, CF14 4XN, UK; 2 South East Wales Trials Unit, Department of Primary Care and Public Health, School of Medicine, Cardiff University. Heath Park, Cardiff, CF14 4XN, UK

## Background

The PAAD Study (Probiotics for Antibiotic Associated Diarrhoea in care homes) involves 2 stages. The first stage is a prospective 12 month observational study to collect data on the amount and type of antibiotic prescribed episodes of Antibiotic Associated Diarrhoea and C*.difficile A*ssociated Diarrhoea and outcome in a randomly selected sample of 9 care homes. The second stage is currently being designed but will take the form of a RCT of probiotic vs. placebo administered alongside the antibiotic. These two studies are governed by separate laws and regulations in relation to mental capacity. PAAD stage 1 is covered by the Mental Capacity Act and, for those service users who lack capacity, personal consultees are able to give agreement to the service user’s participation. PAAD stage 2 is covered by the Medicines for Human Use (Clinical Trials) Regulations and consent is given by a personal legal representative or a professional legal representative of the participant. The PAAD study also presents novel challenges in relation to advanced consent. We propose to explore some of the ethical and practical challenges of conducting these studies (and others like it) within the care home setting using qualitative methods. Our purpose is to optimise the informed consent process in a vulnerable population in preparation for stage two of PAAD.

## Methods

We are conducting focus groups with staff within the 9 care homes who are participating in PAAD stage 1. We are also conducting face-to-face interviews with service users who have capacity, with relatives of service users, and with GPs who provide primary care to the care homes. Focus group and interview schedules focus on issues such as: the merits and problems associated with a number of models of consent for both stage one and stage two of PAAD, views about the feasibility and acceptability of taking advance consent/assent for research trial procedures, and views about potentially raising concerns about the possibility of *c.difficile* circulating within the care home.

## Results

Data collection is in early stages, and we will have data analysed by Autumn 2011.

## Conclusion

We anticipate that our data will not only optimise the design of our informed consent process for stage 2 of PAAD, but will also provide generalisable insights to guide other studies which plan to conduct clinical trials amongst vulnerable populations within care home settings.

